# Deep Learning-Based Liver Tumor Segmentation from Computed Tomography Scans with a Gradient-Enhanced Network

**DOI:** 10.3390/diagnostics16030429

**Published:** 2026-02-01

**Authors:** Hangyeul Shin, Kyujin Han, Seungyoo Lee, Harin Park, Seunghyon Kim, Jeonghun Kim, Xiaopeng Yang, Jae Do Yang, Jisoo Song, Hee Chul Yu, Heecheon You

**Affiliations:** 1 School of Applied Artificial Intelligence and Entrepreneurship, Handong Global University, Pohang 37554, Republic of Korea; 21900408@handong.ac.kr (H.S.); harinnn998@handong.ac.kr (H.P.); 22100128@handong.ac.kr (S.K.); 21800166@handong.ac.kr (J.K.); 2 Graduate School of Artificial Intelligence, Pohang University of Science and Technology, Pohang 37673, Republic of Korea; kyujin@postech.ac.kr; 3 Graduate School of Artificial Intelligence, Korea Advanced Institute of Science and Technology, Daejeon 34141, Republic of Korea; punctuate@kaist.ac.kr; 4 Department of Surgery, Jeonbuk National University Medical School and Hospital, Jeonju 54907, Republic of Korea; hcyu@jbnu.ac.kr; 5 Research Institute of Clinical Medicine, Jeonbuk National University, Jeonju 54907, Republic of Korea; 6 Biomedical Research Institute, Jeonbuk National University Hospital, Jeonju 54907, Republic of Korea; 7 Department of Radiology, Jeonbuk National University Medical School and Hospital, Jeonju 54907, Republic of Korea; pichgo@jbnu.ac.kr; 8 Department of Industrial Management and Engineering, Pohang University of Science and Technology, Pohang 37673, Republic of Korea; hcyou@postech.ac.kr

**Keywords:** deep learning, liver tumor segmentation, computed tomography, gradient-enhanced network

## Abstract

**Background/Objectives**: This study aimed to develop a fully automatic method for liver tumor segmentation based on our previously developed gradient-enhanced network G-UNETR++. **Methods**: The proposed method consists of segmentation of the full liver region from computed tomography (CT) images using G-UNETR++, masking the CT images with the extracted liver region to exclude non-liver regions, and liver tumor segmentation from the masked CT images, also using G-UNETR++. To train and evaluate the model, a total of 131 CT scans (97 for training, 20 for validation, and 20 for testing) from the publicly available LiTS dataset were used. Furthermore, another public dataset, the 3DIRCADb dataset consisting of 20 CT scans was used for cross-validation of the effectiveness and generalizability of our method. **Results**: Experimental results showed that our method outperformed state-of-the-art models over both the LiTS dataset and the 3DIRCADb dataset, with an average dice score of 0.844 and 0.832 over the two datasets, respectively. **Conclusions**: The proposed method is effective in clinical application to help physicians with liver tumor diagnosis and treatment.

## 1. Introduction

Liver cancer ranks as the fourth highest cause of death among all malignancies [1], making its diagnosis and surgical intervention critical tasks in the modern medical field. To enhance the success rates of these procedures, accurate segmentation of both the liver and tumors is essential. Computed tomography (CT) has emerged as a representative imaging modality, widely utilized by physicians for analyzing organs and detecting lesions. In clinical practice, physicians typically detect tumors from a patient’s CT scan through visual observation, relying on their knowledge and experience. This approach is subjective and time-demanding, potentially leading to an increased possibility of misdiagnosis or missed diagnosis [2]. Therefore, automatic and accurate liver tumor segmentation from CT scans is needed to assist physicians in detecting liver tumors efficiently and accurately.

With the advancement of deep learning technologies, numerous methods have been proposed for medical image segmentation tasks. Since the introduction of convolutional neural networks (CNNs) [3], significant progress has been made in the field of computer vision, leading to the development of various CNN-based architectures [4,5,6,7,8,9,10,11,12,13,14,15,16,17]. U-Net [4] has especially become one of the most popular network architectures for medical image analyses tasks. For example, Özcan et al. [17] proposed a hybrid model incorporating U-Net and inception models for automatic segmentation of the liver and liver tumors from CT scans. They reported that their model achieved 75.6% and 65.6% of dice similarity coefficient (*DSC*) over two public datasets, respectively. CNN-based models are able to capture local features but struggle to capture global contexts from medical images due to their localized receptive fields [18].

To address these issues, we aim to develop a two-step method for liver tumor segmentation based on a gradient-enhanced network, referred to as G-UNETR++ [19]. G-UNETR++ is a hybrid network incorporating U-Net, vision transformer (ViT) [20], and gradient-based encoders, originally designed for liver segmentation. ViT is able to capture global contexts by its self-attention mechanisms. Gradient-based encoders further enable G-UNETR++ to learn 3D boundary features of the organs and tissues from medical images. First, the liver is segmented from CT images with G-UNETR++ and the CT images are masked by the extracted liver regions to remove non-liver regions. Then, the regions of liver tumors are delineated with G-UNETR++.

## 2. Related Work

### 2.1. CNN-Based Segmentation Networks

CNN-based architectures have been widely adopted for medical image segmentation, with many approaches derived from U-Net [5,6,10,11,12,13]. Milletari et al. [10] proposed a fully convolutional network, optimized using a dice-based loss for volumetric segmentation. Çiçek et al. [5] extended U-Net to 3D by replacing 2D operations with 3D convolutions, enabling effective volumetric analysis. Dou et al. [11] introduced a deeply supervised 3D network that improved liver segmentation performance on CT volumes. To enhance boundary delineation, Roth et al. [13] proposed a two-stage FCN, achieving higher dice scores in multi-organ CT segmentation. Despite these advances, CNN-based models are limited in capturing long-range dependencies and global contextual information.

### 2.2. Transformer-Based and Hybrid Segmentation Networks

Dosovitskiy et al. [20] introduced attention mechanisms to computer vision, motivating transformer-based medical image segmentation models. Pure transformer architectures without convolution have been proposed for both 2D and 3D segmentation tasks [21,22]. Cao et al. [22] designed a U-Net-like transformer with shifted window attention for encoder–decoder learning, achieving strong results on 2D medical datasets. Karimi et al. [21] introduced a convolution-free 3D model that applies self-attention across neighboring volumetric patches.

To combine local feature extraction with global context modeling, several studies proposed hybrid CNN–transformer architectures [23,24,25,26,27,28,29,30,31]. Oktay et al. [23] enhanced U-Net with attention gates in skip connections, improving abdominal multi-organ segmentation. Valanarasu et al. [24] proposed axial attention for more efficient positional encoding. TransUNet [30] employed a CNN-based encoder followed by transformer blocks and a CNN decoder for precise localization. Similarly, Xie et al. [31] combined CNN feature extraction with transformer-based contextual modeling for 3D segmentation. Hatamizadeh et al. [25] eliminated convolutional encoders and directly tokenized image patches using a transformer, while retaining a skip-connected decoder for multi-scale prediction. Zhou et al. [26] interleaved convolution and self-attention to learn volumetric representations and achieved superior liver segmentation performance on the Synapse dataset compared to UNETR. Shaker et al. [27] further introduced an efficient paired attention block that jointly models spatial and channel dependencies, improving segmentation accuracy and computational efficiency; however, its liver segmentation performance remained slightly lower than nnFormer, and tumor segmentation results were not reported.

### 2.3. Liver and Tumor Segmentation Networks

Several segmentation networks have been specifically designed for liver and tumor segmentation [2,32,33,34,35,36,37,38]. Jin et al. [32] replaced standard U-Net convolutional blocks with residual blocks to enhance feature learning and mitigate gradient vanishing, while integrating multi-scale attention to fuse hierarchical features. Chen et al. [2] proposed MS-FANet, incorporating residual attention blocks and multi-scale atrous downsampling to better capture tumor size and shape variations, achieving dice scores of 0.742 and 0.780 on two public datasets. Jiang et al. [34] introduced RMAU-Net, which integrates squeeze-and-excitation mechanisms and multi-scale feature fusion to model both inter-channel and spatial relationships, achieving dice scores of 0.762 and 0.831 on the same datasets. Muhammad and Zhang [35] leveraged the hybrid ResUNet model, a combination of both the ResNet and UNet models developed by the Monai 0.6 and PyTorch 1.10 frameworks and achieved a dice score of 0.87 for liver tumor segmentation over the public MSD Task03 Liver dataset. Yashaswini et al. [36] applied U-Net and a modified ResUNet to liver and tumor segmentation from CT scans using the 3DIRCADb dataset, demonstrating the effectiveness of fully convolutional networks for semantic segmentation. The ResUNet achieved superior performance, with dice scores of 91.44% for liver segmentation and 75.84% for tumor segmentation. Balaguer-Montero et al. [37] developed a fully automated liver tumor detection and segmentation system based on nnU-Net and achieved a dice score of 81.72% at lesion level. Zhang et al. [38] introduced a novel liver tumor segmentation framework that combines deformable attention, global context modeling, and dual cross-scale feature fusion to handle complex and irregular tumor structures. Their model achieved a dice score of 81.33% on their internal test set. Despite these efforts, accurate tumor segmentation remains challenging due to the heterogeneous appearance and complex morphology of liver tumors.

## 3. Materials and Methods

### 3.1. Data Preparation

Two public CT datasets, including the MICCAI 2017 LiTS dataset (LiTS) [39] and the 3DIRCADb dataset [40], were used in this study. The LiTS dataset is a public dataset from the liver tumor segmentation challenge held at ISBI 2017 and MICCAI 2017. It is the most widely used dataset for liver and tumor segmentation research. The LiTS dataset contains patients with diverse types of liver tumor diseases, consisting of primary tumor disease, such as hepatocellular carcinoma and cholangiocarcinoma, and secondary liver tumors, such as metastases from colorectal, breast, and lung primary cancers. The LiTS dataset, primarily focusing on portal venous phase CT scans, comprises a training set of 131 CT scans and a separate test set of 70 CT scans. The number of CT slices in each scan varies from 42 to 1026, with an axial resolution of 512 × 512 pixels, in-plane voxel dimensions of 0.55 to 1.0 mm and a slice thickness ranging from 0.45 to 6.0 mm. The training dataset was manually labeled by four radiologists from six clinical sites worldwide, whereas labels of the test set are not publicly available. It is challenging to segment tumors using this dataset because of the significant variations in slice thickness, scan image storage direction, image quality, and spatial resolution.

The 3DIRCADb dataset is another publicly available dataset that provides more complex data on the liver and tumors. The 3DIRCADb-01 dataset consists of enhanced CT scans of 10 females and 10 males, with 75% of cases having hepatic tumors, while the 3DIRCADb-02 dataset comprises two 3D CT scans. The voxel dimensions of the dataset are [0.56–0.87, 0.56–0.87, 1.6–4.0] mm, with an axial resolution of 512 × 512 pixels and the number of slices in each scan varying between 74 and 260. In some cases, the liver and tumors have low contrast and overlapping regions, which makes the tumor segmentation a challenging task. Labels of the dataset are publicly available.

In this study, the 131 CT scans from the LiTS dataset were randomly split into 97 cases for training, 20 for validation, and 20 for testing. Next, the 3DIRCADb-01 dataset was used for cross-validation of the effectiveness and generalizability of our method.

### 3.2. Data Preprocessing and Augmentation

**Figure 1:**
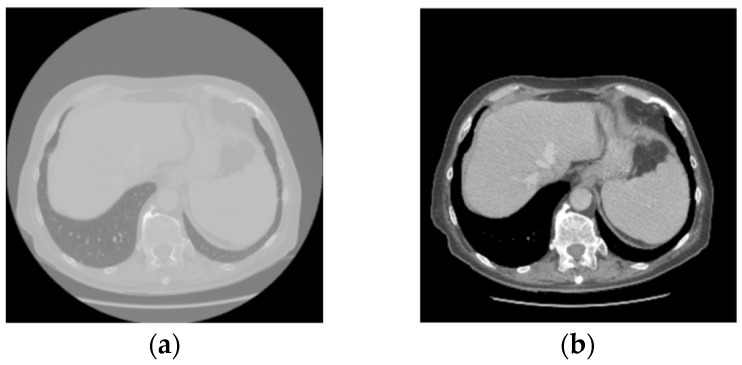
CT image processing performed to enhance the visibility of the liver and tumor regions by normalizing the Hounsfield Unit values to the range of −250 to 250: (**a**) the original CT slice; (**b**) the enhanced CT slice.

Preprocessing was performed to normalize the Hounsfield Unit values of all CT scans to a range of −250 to 250 to enhance the visibility of the liver and tumor regions, as shown in Figure 1. All CT scans were resized from 512 × 512 to 256 × 256 to reduce computational cost. The same data augmentation strategies as G-UNERT++ [19], such as random rotation of 90, 180, and 270 degrees, random scaling, random mirroring, and random intensity shifting were applied.

### 3.3. Deep Learning Model Preparation and Training

**Figure 2:**
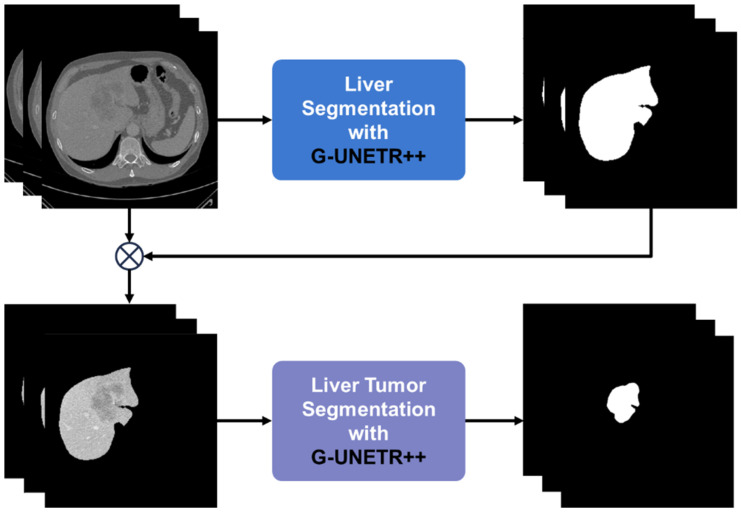
The proposed pipeline for liver tumor segmentation.

**Figure 3:**
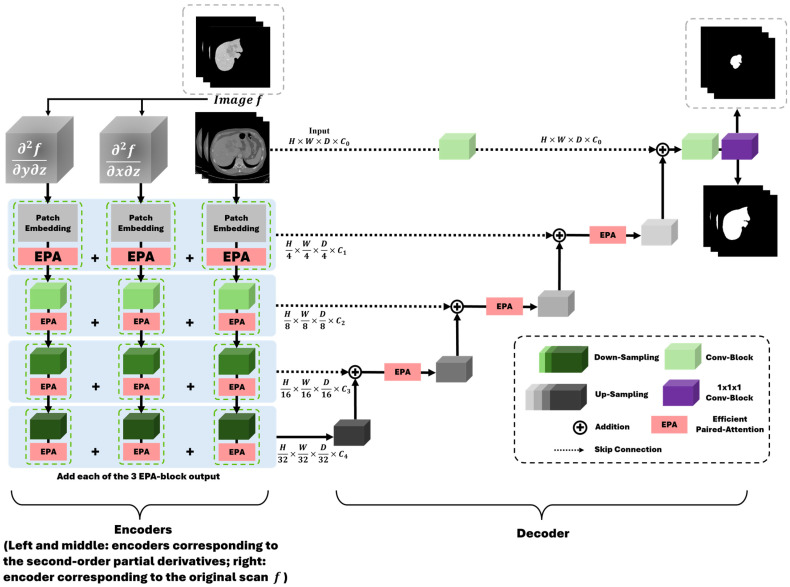
Architecture of G-UNETR++ for liver segmentation and tumor segmentation.

Figure 2 shows the overall pipeline of liver tumor segmentation based on our G-UNETR++ model. First, the liver is segmented from CT images using G-UNETR++. Then the extracted liver region is multiplied with the original CT images to exclude non-liver regions. Lastly, liver tumors are delineated from the liver-only CT images using G-UNETR++. Figure 3 shows the architecture of our G-UNETR++ model. The model consists of a hierarchical encoder–decoder structure with skip connections between the encoder and the decoder, efficient paired attention (EPA) blocks, and convolutional blocks to obtain segmentation results. The encoder scheme consists of three 4-stage encoders in parallel one with CT scan f(x,y,z) as input, and the other two with second-order partial derivatives $\frac{\partial^{2}f(x,y,z)}{\partial x\partial z}$ and $\frac{\partial^{2}f(x,y,z)}{\partial y\partial z}$ as their inputs to learn 3D geometric features such as the boundaries between different organs and tissues especially along the z-axis. In the first stage, patch embedding is performed, where the input volume $f\in R^{H\times W\times D}$ is divided into non-overlapping 3D patches $f_{p}\in R^{N\times {(P}_{1},{\;P}_{2},{\;P}_{3})}$ of size (*P*_1_, *P*_2_, *P*_3_), producing a sequence with length $N=\left(\frac{H}{P_{1}}\times \frac{W}{P_{2}}\times \frac{D}{P_{3}}\right)$. These patches are projected into $C_{1}$ channels to form a $\frac{H}{P_{1}}\times \frac{W}{P_{2}}\times \frac{D}{P_{3}}\times C_{1}$ feature map, using a patch resolution of (4, 4, 2), followed by an EPA block. The remaining stages downsample the feature maps by a factor of two using non-overlapping convolutions, each followed by an EPA block. At every stage, features from the three encoders are fused via element-wise summation. The EPA block employs spatial and channel attention with shared keys and queries and separate value projections to jointly model spatial–channel features [27]. The decoder consists of four stages with deconvolution-based upsampling to progressively increase resolution. EPA blocks are used at all but the final stage. Skip connections fuse encoder and decoder features at each scale to recover spatial information, while channel dimensions are halved between stages. In the final stage, fused features are passed through 3 × 3 × 3 and 1 × 1 × 1 convolutions to generate voxel-wise segmentation outputs.

### 3.4. Loss Function

We proposed different hybrid loss functions for liver segmentation and tumor segmentation. For liver segmentation, a hybrid loss function $L_{liver}$ was proposed, consisting of dice loss $L_{dice}$, cross-entropy (CE) loss $L_{CE}$, and Hausdorff distance (HD) loss [41] $L_{HD}$. For tumor segmentation, a hybrid loss function $L_{tumor}$ was proposed, consisting of dice loss, focal loss [42] $L_{focal}$, and HD loss to address the class imbalance issue in tumor segmentation. The hybrid loss function for liver segmentation is defined as follows:

(1) $$L_{liver}=\alpha_{1}L_{dice}+{\beta_{1}L}_{CE}+{\gamma_{1}L}_{HD}$$

where $\alpha_{1}=\beta_{1}=\gamma_{1}=1$. The hybrid loss function for tumor segmentation is defined as follows:

(2) $$L_{tumor}=\alpha_{2}L_{dice}+{\beta_{2}L}_{focal}+{\gamma_{2}L}_{HD}$$

where $\alpha_{2}=\beta_{2}=\gamma_{2}=1$. Dice loss is defined as follows:

(3) $$L_{Dice}=\;1-\;\frac{2*\;\sum_{i=1}^{N}p_{i}y_{i}}{\sum_{i=1}^{N}p_{i}^{2}+\sum_{i=1}^{N}y_{i}^{2}}$$

where N denotes the number of predicted voxels; $p_{i}$ indicates the prediction probability at voxel i; and $y_{i}$ represents the ground truth at voxel i. CE loss is defined as follows:

(4) $$L_{CE}\left(y,\;p\right)=\left\{\begin{array}{ll} -\log p & ,\;\;y=1\\ -\log(1-p) & ,\;\;y=0 \end{array}\right.$$

where y indicates the ground truth and p denotes the prediction probability. HD loss is defined as follows:

(5) $$L_{HD}=\;\underset{x\in X}{\max}\;\underset{y\in Y}{\min}{\left\|x-y\right\|}_{2}$$

where x∈X denotes the predicted binary segmentation with a threshold of 0.5 and y∈Y indicates the ground truth. Focal loss is defined as follows:

(6) $$L_{focal}\left(p_{t}\right)=-a_{t}{\left(1-p_{t}\right)}^{\gamma }log(p_{t})$$

where $p_{t}$ denotes the predicted probability for the correct class; $a_{t}=1$ indicates a weighting factor for class imbalance; and γ=2 denotes the focusing parameter, controlling the rate at which easy examples are downweighted. A deep supervision technique [43] was applied into our decoder for better training efficiency.

### 3.5. Model Training

Identical settings were applied to train both the liver segmentation model and the tumor segmentation model. All experiments were performed with an NVIDIA RTX 3090 GPU. The initial learning rate was set to 5 × 10^−4^ with a poly decay strategy [26]. The Adam optimizer [44] was used with a weight decay of 3 × 10^−5^. The number of epochs was set as 1000. The batch size was set as 8.

### 3.6. Post-Processing

By visual inspection, we observed that our model tended to segment tumors with fewer voxels compared to the ground truth, even though the model accurately predicted the locations of the tumors. This undersizing of small tumors became more severe as the size of the tumor decreased in the ground truth. To address this issue, we empirically applied the morphological dilation method twice, with a pixel size of one in each operation to the predicted tumor regions smaller than 100 pixels from a CT slice after exhaustive experimentation.

### 3.7. Evaluation Metrics

To assess the performance of the proposed tumor segmentation method, we utilized a range of evaluation metrics, including the dice similarity coefficient (*DSC*) to measure overlap accuracy, volumetric overlap error (*VOE*) to measure volume consistency, relative absolute volume difference (*RAVD*) to assess volume disparity, and average symmetric surface distance (*ASSD*) to quantify surface deviation.

*DSC* quantifies the overlap between the predicted volumetric output (*Pred*) and the ground truth (*GT*). Ranging from 0 to 1, *DSC* = 1 indicates perfect overlap and segmentation, while *DSC* = 0 signifies no overlap between the predicted and the ground truth. The formula for *DSC* is as follows:

(7) $$DSC=\frac{2|Pred\;\cap \;GT|}{\left|Pred\right|+\;|GT|}$$

*VOE* quantifies the alignment between the predicted and the ground truth, assessing the error rate in segmentation. Ranging from 0 to 1, *VOE* = 0 indicates perfect overlap, while *VOE* = 1 signifies no overlap. The formula for *VOE* is as follows:

(8) $$VOE=1-\;\frac{\left|\;Pred\;\cap \;GT\;\right|}{\left|\;Pred\;\cup GT\;\right|}$$

*RAVD* is used to measure the volume discrepancy between the predicted and the ground truth, ranging from 0 to 1, with a value of zero indicating no volume disparity, reflecting a perfect segmentation. *RAVD* is calculated as follows:

(9) $$RAVD=\left|\frac{\left|\;Pred\;\left|\;-\;\right|\;GT\;\right|}{\left|\;GT\;\right|}\;\right|$$

*ASSD* is the key metric for measuring the average deviation between the surfaces of the predicted and the ground truth, with a value of zero indicating a perfect segmentation. The shortest distance of a voxel x to the set of surface voxels S(GT) of GT is defined as follows:

(10) $$d\left(x,S\left(GT\right)\right)=\underset{y\in S(\mathrm{GT})}{\min}\left\|x-y\right\|$$

where ‖x−y‖ denotes the Euclidean distance between x and y. Then *ASSD* is defined as follows:

(11) $$ASSD=\frac{1}{\left|S\left(Pred\right)\right|+\left|S\left(GT\right)\right|}\times \left(\sum_{x\in S\left(Pred\right)}d\left(x,\;S\left(GT\right)\right)+\sum_{y\in S\left(GT\right)}d\left(y,S\left(Pred\right)\right)\right)$$

## 4. Results

We compared our model with state-of-the-art models for liver tumor segmentation, including HDU-Net [45], ResUNet [46], MS-FANet [2], HFRU-Net [47], and RMAU-Net [34]. Table 1 shows the comparison results for liver tumor segmentation over the LiTS and 3DIRCADb datasets. For both datasets, our model outperformed state-of-the-art models. For the LiTS dataset, our model showed a significant improvement in liver tumor segmentation compared with the other models. Our model achieved the best performance in terms of *DSC* (0.844), *VOE* (0.263), and *ASSD* (1.317 mm), with *RAVD* being the only exception, which indicates the high effectiveness of our model in accurately capturing tumor regions. Next, for the 3DIRCADb dataset, our model also achieved the best performance in terms of *DSC* (0.832) and *ASSD* (1.682), except for *VOE* and *RAVD*. The consistently high performance of our model across different datasets highlights its robustness and generalizability.

**Figure 4:**
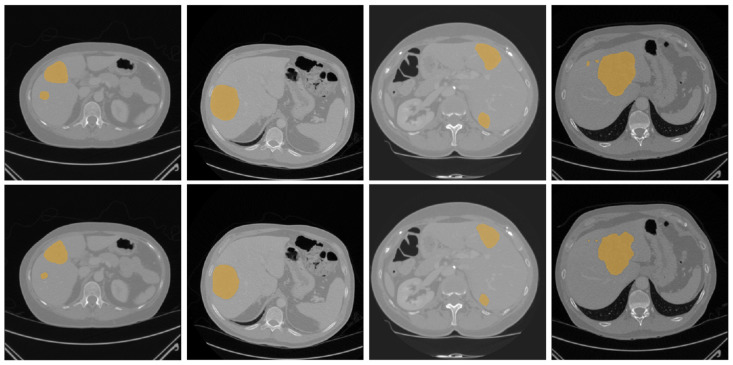
Examples of tumor segmentation results of the proposed model over the LiTS dataset. **Upper**: the ground truth; **bottom**: the predicted tumor regions.

**Figure 5:**
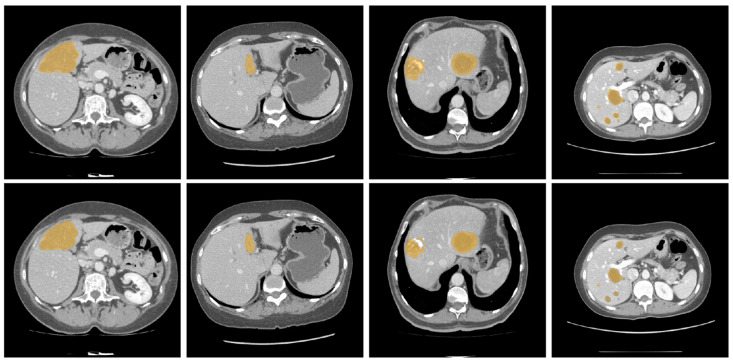
Examples of tumor segmentation results of the proposed model over the 3DIRCADb dataset. **Upper**: the ground truth; **bottom**: the predicted tumor regions.

Figure 4 and Figure 5 illustrate some examples of the liver tumor segmentation results with our model from the LiTS dataset and the 3DIRCADb dataset, respectively. Visual inspection of the segmentation results reveals that the predicted tumor regions are properly aligned with the ground truth in terms of both the number of tumors and their morphology.

We conducted an ablation study to demonstrate the effectiveness of the proposed post-processing method. First, we evaluated the post-processing method on the LiTS and 3DIRCADb datasets. As shown in Table 2, for the LiTS dataset, there is a slight performance difference between the results with post-processing and those without post-processing, whereas for the 3DIRCADb dataset, the dice score has been improved by 2.9% and all other metrics have also been improved. Therefore, the proposed post-processing method is effective in improving tumor segmentation results.

**Figure 6:**
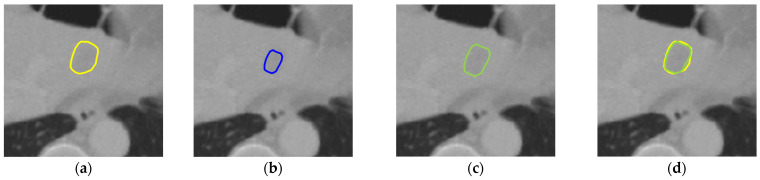
An illustration of the post-processing result: (**a**) the ground truth tumor boundary (yellow); (**b**) the predicted tumor boundary before post-processing (blue); (**c**) the dilated tumor boundary after post-processing (green); (**d**) the overlay of the ground truth tumor boundary (yellow) and the dilated tumor boundary (green).

To determine the right number of times of the morphological dilation operation in our post-processing method, we applied the morphological dilation operation from one to three times to compare their performance. As shown in Table 3, for the LiTS dataset, the best performance was achieved with the one-time application of the dilation operation, but the differences with the two-time application and the three-time application were small, whereas for the 3DIRCADb dataset, the results showed that applying dilation twice resulted in the best performance in terms of all evaluation metrics. Therefore, the post-processing method that applies the morphological dilation twice was adopted for our liver tumor segmentation method. As shown in Figure 6, after post-processing, the dilated tumor boundary is closer to the ground truth tumor boundary.

**Figure 7:**
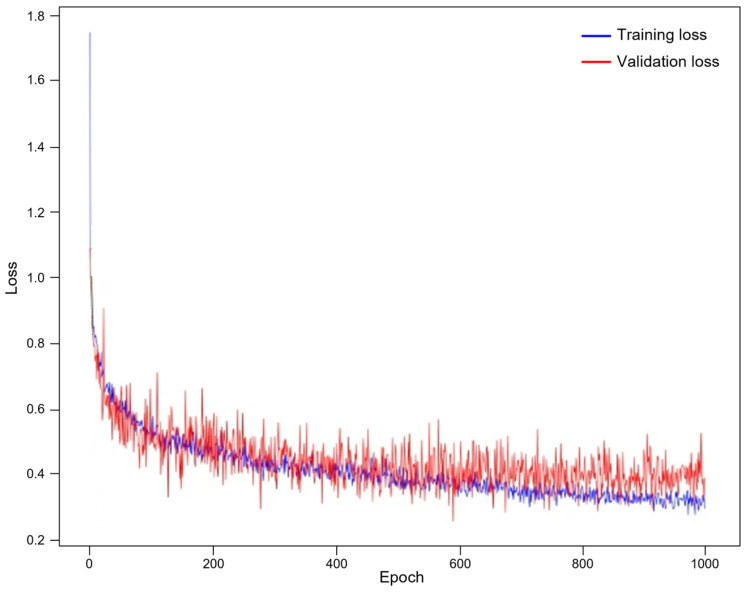
Plotting of training loss and validation loss during our model development.

**Figure 8:**
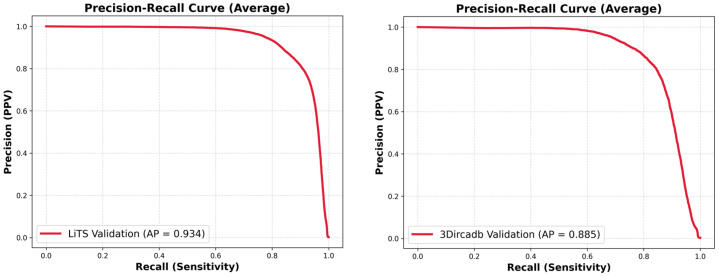
Precision-recall plotting for liver tumor segmentation over the LiTS dataset (**left**) and the 3DIRCADb dataset (**right**).

We conducted various analyses to further evaluate our model. Figure 7 shows the plotting of the training loss and validation loss, indicating that no overfitting occurred during our model development process. Table 4 summarizes the complexity of our model. Figure 8 presents the precision-recall plotting of our model over the LiTS dataset and the 3DIRACDb dataset, respectively.

## 5. Discussion

This study proposed a solid deep learning method for liver tumor segmentation based on G-UNETR++, a gradient-enhanced network originally developed in our previous study for liver segmentation from CT images. The proposed method consists of two steps: (1) segmentation of the liver from a CT scan by G-UNETR++ and masking the CT scan with the extracted liver region to exclude non-liver regions, and (2) segmentation of tumors from the masked CT scan by G-UNETR++.

Performance comparisons on the LiTS dataset show that our method outperforms existing state-of-the-art methods, achieving an average *DSC* of 0.844. To assess generalizability, the model was evaluated on one unseen dataset, 3DIRACDb, where it achieved an average *DSC* of 0.832. These results demonstrate strong robustness across datasets acquired under diverse conditions. The inclusion of two gradient-enhanced encoders ensures that our model effectively captures 3D geometric features. Furthermore, the proposed hybrid loss function can handle the class imbalance issue with focal loss and deal with difficult cases in tumor segmentation to ensure boundary precision with HD loss. Lastly, the post-processing method reduces tumor segmentation error by morphological dilation operation, especially when the tumor size is small.

**Figure 9:**
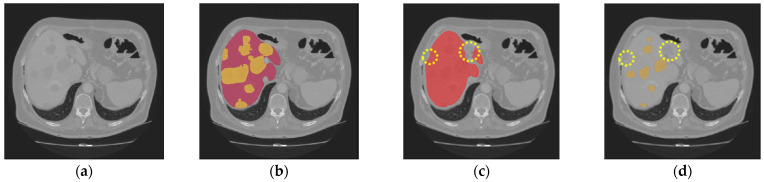
The following are missing in the final tumor segmentation results due to the missing tumors in liver segmentation results: (**a**) the original CT slice; (**b**) the ground truth for the liver (red) and tumors (orange); (**c**) the liver segmentation result with two tumors missing (highlighted in dotted circles); (**d**) the tumor segmentation results (orange) with two tumors missing (highlighted in dotted circles).

**Figure 10:**
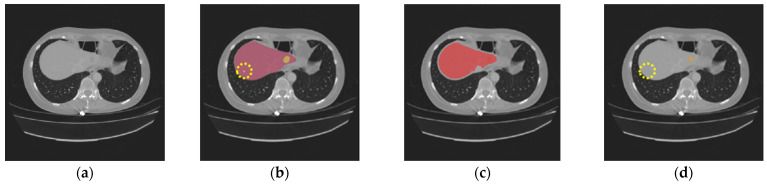
An extremely small tumor missing in the final tumor segmentation results: (**a**) the original CT slice; (**b**) the ground truth for the liver (red) and tumors (orange) with an extremely small tumor (highlighted in a dotted circle); (**c**) the liver segmentation result; (**d**) the tumor segmentation result (orange) with one tumor missing (highlighted in a dotted circle).

Nevertheless, our study has limitations in certain aspects. The introduction of the two gradient-enhanced encoders increases the complexity level of our model and therefore the computational cost. Furthermore, the tumor segmentation performance can be affected by the liver segmentation performance. The proposed method achieved high performance on both the LiTS dataset and the 3DIRCADb dataset, with an average *DSC* of 97.38% and 97.50% for liver segmentation, respectively. However, in some cases, tumors may be missing in the segmented liver region, as shown in Figure 9c. Then, in the masking step, those tumors will be excluded from the CT images masked by the extracted liver region, which will be used as the input for tumor segmentation. In that case, those tumors will be missing in tumor segmentation, as shown in Figure 9d. In addition, our model may occasionally miss segmenting some extremely small tumors, as shown in Figure 10. This could be caused by tumor size imbalance and biased annotation for small lesions in the public datasets used for our model development. For our future work, the proposed two-step liver tumor segmentation pipeline can be reduced to a single-step pipeline by applying an end-to-end framework. This would enable a direct extraction of tumors from the original CT scans, thereby reducing computational costs and potentially enhancing the robustness of the segmentation process. Furthermore, the sizes of the datasets used for model training and evaluation are relatively small. To strengthen the experimental validity, the performance of the proposed model needs to be further evaluated using k-fold cross-validation or a larger dataset. Lastly, the ablation study is limited in scope and primarily focuses on the effect of post-processing. Though the ablation study in our previous work [19] concluded that the inclusion of the proposed gradient-enhanced encoders and a hybrid loss function that incorporates the HD loss are effective in improving the performance of liver segmentation, their effects on liver tumor segmentation were not studied in the current study. For future work, a comprehensive ablation study that analyzes the impacts of the gradient-enhanced encoders, the proposed hybrid loss function, and the EPA blocks on improving liver tumor segmentation performance will be conducted.

## 6. Conclusions

This study presents a deep learning framework for liver tumor segmentation from CT images using a two-step pipeline consisting of liver extraction followed by tumor segmentation. The proposed method achieves competitive performance, outperforming existing approaches on the LiTS dataset with a *DSC* of 0.844, and demonstrating strong generalizability on the unseen 3DIRACDb dataset with a *DSC* of 0.832.

The incorporation of gradient-enhanced encoders enables the effective learning of 3D geometric features, while the hybrid loss function addresses class imbalance and improves boundary delineation. In addition, post-processing with morphological dilation reduces segmentation errors, particularly for small tumors. Despite these advantages, the model’s complexity and reliance on accurate liver segmentation remain limitations, and very small tumors may still be missed.

Future work will focus on developing an end-to-end, single-stage framework to reduce computational cost and improve robustness, as well as validating the method using k-fold cross-validation or larger datasets with comprehensive ablation studies.

The proposed method shows strong clinical potential by accurately segmenting liver tumors from CT scans. The generated segmentation results allow radiologists to efficiently review tumor regions and assess imaging characteristics for diagnosis, supporting informed treatment planning by physicians.

## Figures and Tables

**Table 1:** A comparison of the proposed model with state-of-the-art models for liver tumor segmentation over the LiTS and 3DIRCADb datasets.

Dataset	Method	***DSC***	***VOE***	***RAVD***	***ASSD***
LiTS	HDU-Net [45]	0.711	0.401	0.023	7.201
	ResUNet [46]	0.705	0.395	0.534	8.286
	MS-FANet [2]	0.742	0.367	0.107	5.996
	HFRU-Net [47]	0.749 ± 0.107	0.380 ± 0.128	0.218 ± 0.152	-
	RMAU-Net [34]	0.762 ± 0.118	0.371 ± 0.135	0.012 ± 0.291	-
	The proposed	0.844 ± 0.078	0.263 ± 0.114	0.133 ± 0.143	1.317 ± 0.645
3DIRCADb	HDU-Net	0.692	0.382	4.835	16.516
	ResUNet	0.739	0.357	0.102	7.817
	MS-FANet	0.780	0.313	0.155	5.346
	HFRU-Net	0.789 ± 0.111	0.326 ± 0.142	0.033 ± 0.170	-
	RMAU-Net	0.831 ± 0.095	0.275 ± 0.125	0.126 ± 0.186	-
	The proposed	0.832 ± 0.060	0.283 ± 0.085	0.138 ± 0.111	1.682 ± 1.029

**Table 2:** The ablation study conducted to evaluate the performance of the proposed post-processing method in liver tumor segmentation with the LiTS and 3DIRCADb datasets.

Dataset	Method	***DSC***	***VOE***	***RAVD***	***ASSD***
LiTS	Without post-processing	0.845	0.261	0.143	1.267
	With post-processing	0.844	0.263	0.133	1.317
3DIRCADb	Without post-processing	0.803	0.313	0.198	1.784
	With post-processing	0.832	0.283	0.138	1.682

**Table 3:** The ablation study conducted to determine the right number of dilation times for the proposed post-processing method in liver tumor segmentation with the LiTS and 3DIRCADb datasets.

Dataset	Dilation Times	***DSC***	***VOE***	***RAVD***	***ASSD***
LiTS	1	0.845	0.261	0.138	1.311
	2	0.844	0.263	0.133	1.317
	3	0.841	0.267	0.127	1.327
3DIRCADb	1	0.827	0.291	0.164	1.712
	2	0.832	0.283	0.138	1.682
	3	0.824	0.294	0.192	1.693

**Table 4:** A summary of the complexity level of the proposed model.

Number of Parameters	Floating Point Operations/Second	Training Time/Epoch	Inference Time/CT Scan	
			LiTS Dataset	3DIRACDb Dataset
97.73 M	73.12 G	26 min	176.9 ± 124.8 s	43.8 ± 12.8 s

## Data Availability

The raw data supporting the conclusions of this article will be made available by the authors on request.
